# Supplementary material for: Novel Driver Strength Index highlights important cancer genes in TCGA PanCanAtlas patients
Source: PeerJ. 2022 Aug 11;10:e13860. doi: 10.7717/peerj.13860 (PMC9375969; doi:10.7717/peerj.13860)
Supplement: Supplemental Information 2 [file peerj-10-13860-s002.zip › COHORTS/genes plots/cohorts/2021_8_16_14_9_distribution_cohorts_males_PAAD.pdf]

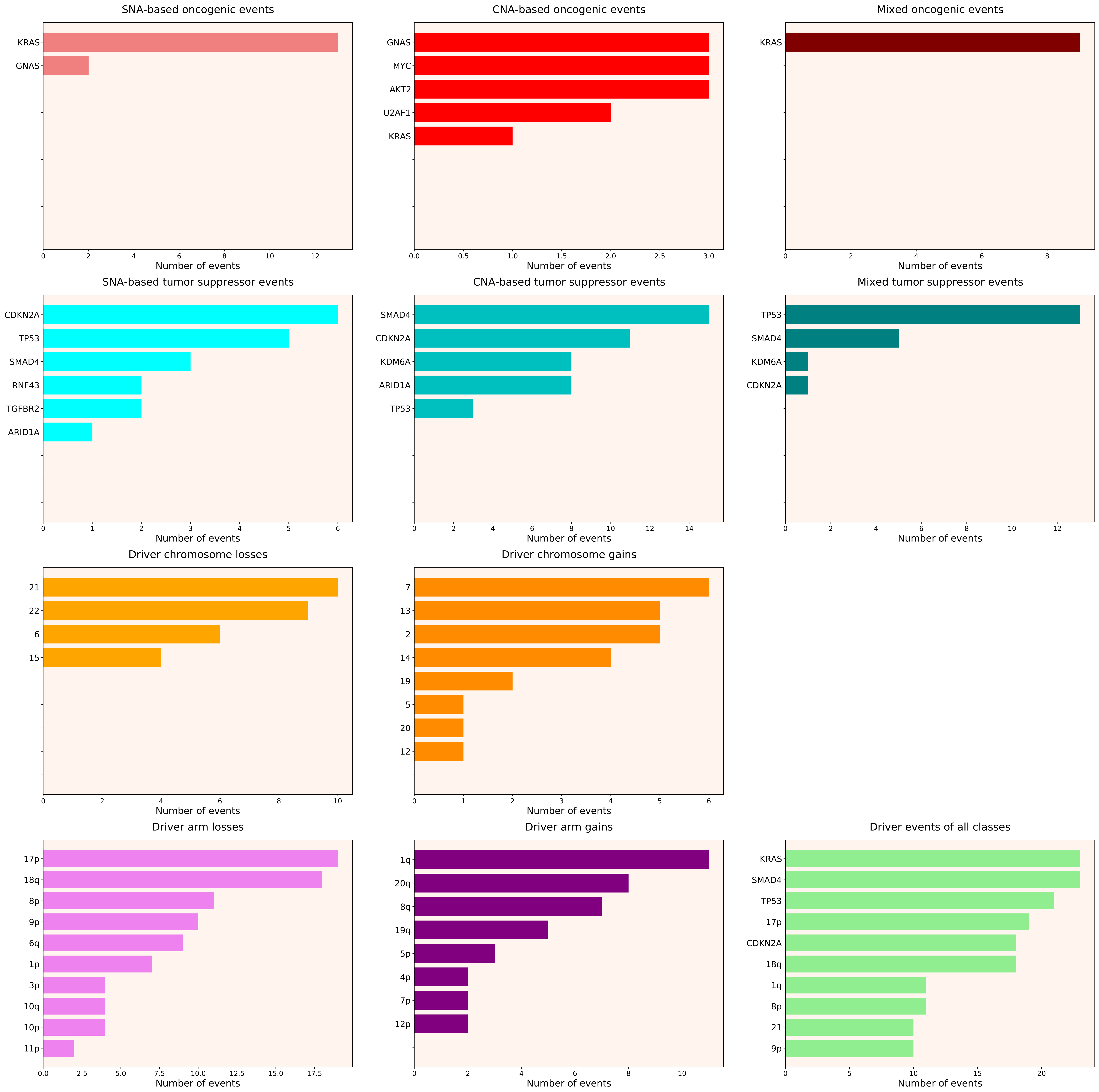

SNA-based oncogenic events
KRAS
GNAS
0 2 4 6 8 10 12
Number of events
CNA-based oncogenic events
GNAS
MYC
AKT2
U2AF1
KRAS
0.0 0.5 1.0 1.5 2.0 2.5 3.0
Number of events
Mixed oncogenic events
KRAS
0 2 4 6 8
Number of events
SNA-based tumor suppressor events
CDKN2A
TP53
SMAD4
RNF43
TGFBR2
ARID1A
0 1 2 3 4 5 6
Number of events
CNA-based tumor suppressor events
SMAD4
CDKN2A
KDM6A
ARID1A
TP53
0 2 4 6 8 10 12 14
Number of events
Mixed tumor suppressor events
TP53
SMAD4
KDM6A
CDKN2A
0 2 4 6 8 10 12
Number of events
Driver chromosome losses
21
22
6
15
0 2 4 6 8 10
Number of events
Driver chromosome gains
7
13
2
14
19
5
20
12
0 1 2 3 4 5 6
Number of events
Driver arm losses
17p
18q
8p
9p
6q
1p
3p
10q
10p
11p
0.0 2.5 5.0 7.5 10.0 12.5 15.0 17.5
Number of events
Driver arm gains
1q
20q
8q
19q
5p
4p
7p
12p
0 2 4 6 8 10
Number of events
Driver events of all classes
KRAS
SMAD4
TP53
17p
CDKN2A
18q
1q
8p
21
9p
0 5 10 15 20
Number of events
